# Suspected Acute Compartment Syndrome Following Snakebite Envenomation: A Fatal Case Highlighting the Conflict Between Life-Saving Resuscitation and Limb-Saving Fasciotomy

**DOI:** 10.7759/cureus.115002

**Published:** 2026-08-22

**Authors:** Darius Mbina Nzienghi, Rachad Bouya, Liela Agnès Okoyi Ossouka, Shamenta Walène Ondo, Said Jidane, Tahir Nebhani, Naoufal Chouaib, Ahmed Belkouch, Lahcen Belyamani

**Affiliations:** 1 Emergency Department, Mohamed V Military Training Hospital, Rabat, MAR; 2 Emergency Department, Mohammed V University, Rabat, MAR; 3 Emergency Department, Université des Sciences et Techniques de la Santé, Libreville, GAB

**Keywords:** acute compartment syndrome, emergency medicine, fasciotomy, snakebite, snake venom, upper extremity trauma

## Abstract

Acute compartment syndrome (ACS) is a rare but devastating potential complication of snake envenomation. Distinguishing suspected ACS from venom-induced pseudo-compartment syndrome remains a major clinical challenge, as unnecessary fasciotomy in a coagulopathic patient carries substantial risks, whereas delayed decompression may result in irreversible ischemic injury.

We report the case of a 68-year-old man admitted 48 hours after a snakebite with massive progressive swelling of the left upper limb, profound metabolic acidosis, severe consumptive coagulopathy, and refractory shock. Surgical decompression was strongly considered due to high clinical suspicion of ACS; however, emergency fasciotomy could not be safely performed owing to severe hemodynamic instability and uncorrected coagulopathy. Despite aggressive resuscitation, antivenom administration, and hemostatic support, the patient rapidly deteriorated and developed refractory cardiac arrest, culminating in death 90 minutes after admission in the setting of overwhelming systemic envenomation.

This case highlights both the diagnostic uncertainty surrounding ACS without access to intracompartmental pressure monitoring and the critical therapeutic dilemma of managing severe local complications alongside life-threatening systemic toxicity.

## Introduction

Snakebite envenoming is a major global public health problem and is recognized by the World Health Organization as a neglected tropical disease [1]. In Morocco, snakebites remain an important health concern, particularly in rural and remote areas where delayed presentation and limited access to specialized care may contribute to poor outcomes [2]. Although most envenomed patients develop local manifestations such as pain, edema, and tissue injury, severe systemic complications, including coagulopathy, acute kidney injury, neurotoxicity, hemorrhage, and circulatory shock, may occur and are associated with substantial morbidity and mortality [3,4].

Among local complications, acute compartment syndrome (ACS) is a rare but potentially limb-threatening surgical emergency caused by increased intracompartmental pressure leading to impaired tissue perfusion [5]. Distinguishing true ACS from venom-related local tissue effects remains challenging, particularly in resource-limited settings where intracompartmental pressure monitoring may be unavailable [6,7].

We report a fatal case of suspected ACS following snakebite envenomation in a patient presenting with profound circulatory shock and severe coagulopathy. This case highlights the diagnostic uncertainty surrounding ACS after snakebite and the complex therapeutic decision-making required when limb-threatening local complications coexist with life-threatening systemic envenomation.

## Case presentation

A 68-year-old man with no prior medical history presented to our emergency department 48 hours after sustaining a snakebite to the volar aspect of his left wrist. Immediately following the bite, he experienced severe localized pain and rapidly spreading edema of the hand. He initially sought traditional local remedies before presenting for care. Over the subsequent 24 to 36 hours, the pain worsened significantly, accompanied by progressive paresthesia, finger numbness, and edema extending proximally to the shoulder. The subsequent onset of systemic manifestations-including severe dizziness, dark urine, nausea-finally prompted emergency transfer to our tertiary center. The offending snake species could not be identified.

Upon admission, he was hypotensive (76/42 mmHg) and tachycardic (118 bpm), with an oxygen saturation of 98% on room air and a body temperature of 37.2 °C. Physical examination of the affected limb revealed massive, tense, shiny, circumferential edema involving the entire left upper limb. He reported severe pain that was disproportionate to the apparent clinical findings and significantly worsened by passive finger extension. Diffuse paresthesia predominantly affecting the distal extremity was noted. The left hand was cold, the radial pulse was barely palpable, capillary refill time exceeded 4 seconds, and the limb exhibited diffuse skin mottling. Two pinpoint fang marks, with minimal oozing and surrounded by an erythematous halo, were visible on the volar aspect of the left wrist (Figure 1).

**Figure 1:**
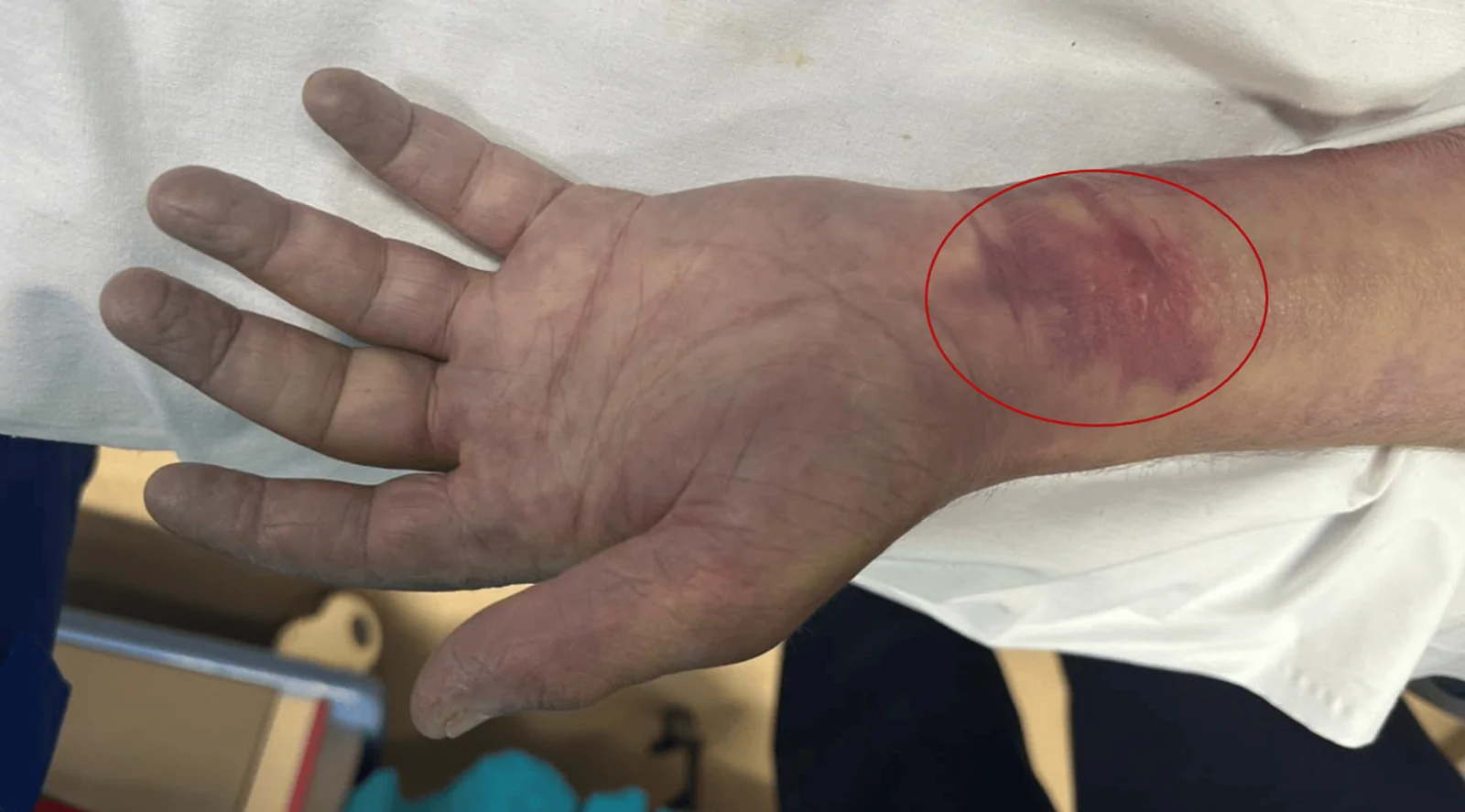
Initial local examination of the left wrist upon admission. Volar aspect of the left wrist demonstrating the bite site (red circle), characterized by two faint pinpoint fang marks, localized ecchymosis, minimal oozing, and a surrounding erythematous halo. Note the marked swelling and cyanosis extending into the hand and fingers.

Initial laboratory investigations revealed several major abnormalities, summarized in Table 1.

**Table 1:** Laboratory findings on admission aPTT: Activated partial thromboplastin time, AST: Aspartate aminotransferase, ALT: Alanine aminotransferase. Laboratory results show hypofibrinogenemia, prolonged prothrombin time, elevated aPTT ratio and D-dimer, elevated creatine kinase and transaminases, and elevated urea and creatinine.

Parameter	Patient’s result	Normal range
White blood cell count (×10^9^/L)	4.6	4.0-10.0
Neutrophils (%)	79.5	40-75
Lymphocytes (%)	10	20-45
Eosinophils (%)	3.1	0-6
Red blood cell count (10^12^/L)	6.18	3.9-5.5
Hemoglobin (g/dL)	17.7	13.0-17.0
Platelet count (×10^9^/L)	180	150-450
Prothrombin time (%)	31	70-100
aPTT ratio	1.5	<1.2
C-reactive protein (mg/L)	13	<5
Cardiac troponin (ng/L)	28	2-34
Sodium (mmol/L)	134	135-145
Potassium (mmol/L)	5.21	3.5-5.5
Chloride (mmol/L)	103	95-110
Urea (mmol/L)	17.5	2.5-6.7
Creatinine (µmol/L)	354	64-104
AST (U/L)	117	<35
ALT (U/L)	73	<40
Total bilirubin (mg/L)	27	3-10
Creatine kinase (U/L)	2132	24-195
Fibrinogen (g/L)	0.4	2-4
D-dimer (µg/L)	2381	<500

Blood gas analysis demonstrated profound metabolic acidosis with a pH of 6.80 (normal: 7.35-7.45), serum bicarbonate of 3.2 mmol/L (normal: 18-28 mmol/L), PaCO₂ of 20.2 mmHg (normal: 35-45 mmHg), and a lactate level of 13.2 mmol/L (normal: <2 mmol/L).

Arterial and venous Doppler ultrasonography of the left upper limb evaluated the brachial, radial, and ulnar arteries, as well as the deep and superficial venous system. The examination demonstrated high-resistance, low-velocity dampened waveforms in both the radial and ulnar arteries, without evidence of acute arterial thrombosis, luminal occlusion, or deep venous thrombosis. Unfortunately, the original Doppler images were not archived in the radiology database and were therefore unavailable for inclusion; findings are reported based on the contemporaneous signed radiology report documented in the patient’s medical record.

ACS of the left upper limb was strongly suspected based on the clinical, vascular, and laboratory findings, in association with clinical signs of distal limb hypoperfusion, rhabdomyolysis, severe venom-induced consumptive coagulopathy (VICC), and venom-induced shock, all occurring in the setting of severe, life-threatening systemic envenomation.

Medical management was immediately initiated in the resuscitation room. Hemodynamic support consisted of cautious volume expansion with isotonic crystalloids and continuous norepinephrine infusion. Given the severity of the envenomation, antivenom therapy was initiated within 15 minutes of admission; a total initial dose of four vials (10 mL per vial) of polyvalent equine F(ab’)₂ antivenom (Inoserp® MENA, Inosan Biopharma, Madrid, Spain) was administered as a continuous intravenous infusion diluted in 250 mL of 0.9% normal saline. Concurrently with antivenom administration, aggressive hemostatic resuscitation was initiated to treat severe VICC and limit microvascular bleeding risk. Given the profound hypofibrinogenemia (0.4 g/L) and prolonged prothrombin time (31%), replacement therapy comprised 2 g of fibrinogen concentrate IV, 1750 IU of prothrombin complex concentrate IV (25 IU/kg), and 4 units of fresh frozen plasma (approximately 15 mL/kg). Critical metabolic disturbances were managed with intravenous sodium bicarbonate in the setting of profound metabolic acidosis, together with treatment for hyperkalemia. Hydrocortisone (200 mg IV) and intravenous H1-antihistamines were given prior to antivenom infusion as premedication against early adverse reactions to equine F(ab’)₂ antivenom. Empirical antibiotic therapy with amoxicillin-clavulanic acid (2g IV) was initiated due to the risk of bacterial contamination from local traditional herbal remedies applied prior to presentation.

In parallel, an urgent joint evaluation was conducted by the emergency medicine, intensive care, and vascular surgery teams. While surgical decompression was strongly considered due to high clinical suspicion of compartment syndrome, the multidisciplinary team decided to defer fasciotomy due to prohibitive perioperative risks from refractory shock and severe consumptive coagulopathy.

Despite the immediate implementation of these intensive measures, the patient’s condition rapidly deteriorated. Thirty minutes after admission, edema of the affected limb worsened, peripheral pulses became impalpable, ecchymoses and marked cyanosis of the hand developed, and mottling rapidly spread to the entire body (Figure 2).

**Figure 2:**
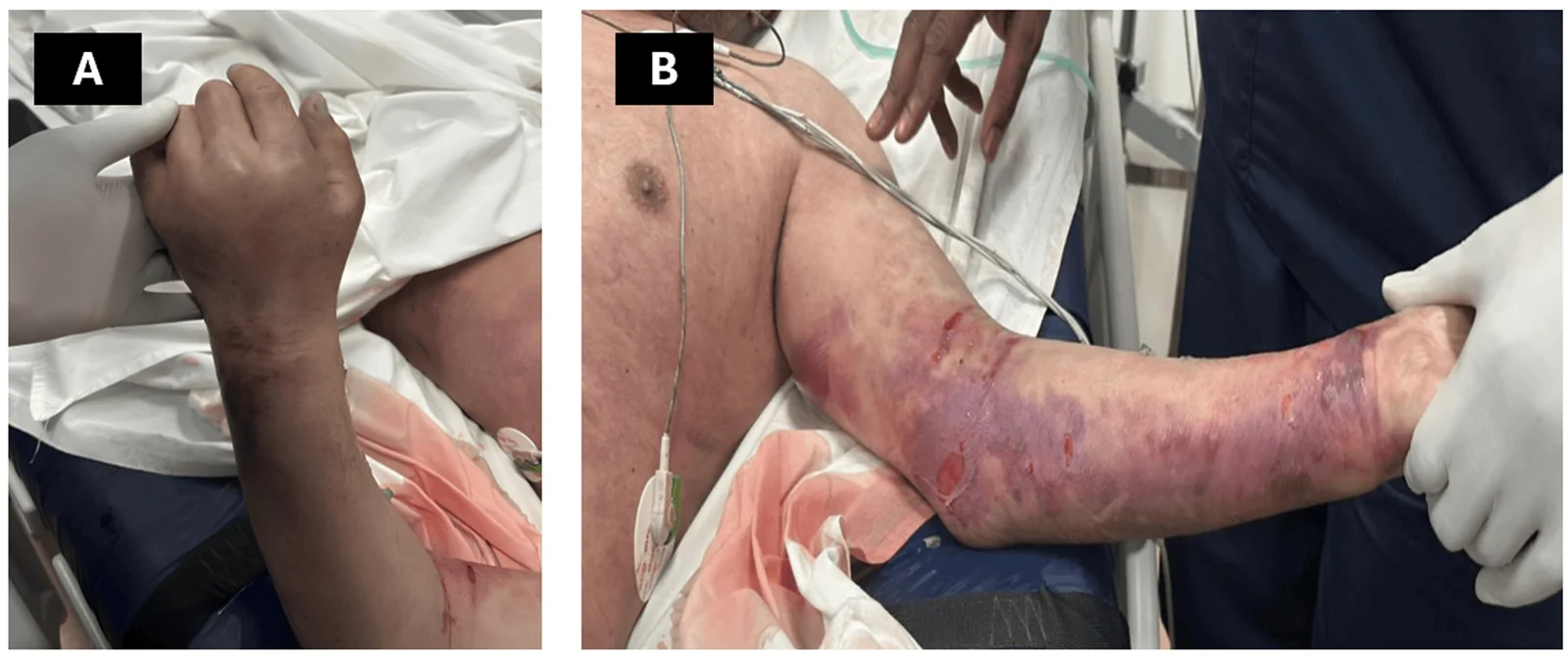
Clinical appearance of the left upper limb 30 minutes after hospital admission. Clinical photographs obtained 30 minutes after hospital admission. (A) Marked swelling of the left hand with tense edema and clinical signs of distal ischemia. (B) Extensive edema involving the entire left upper limb with diffuse ecchymosis and hemorrhagic skin lesions.

Respiratory and neurological status progressively deteriorated as the patient developed acute respiratory distress (respiratory rate: 35 breaths/min; oxygen saturation: 90%) associated with an agitated confusional state in the setting of hemodynamic instability, necessitating rapid-sequence endotracheal intubation. Approximately 45 minutes after admission, the patient developed cardiac arrest. Despite 45 minutes of advanced cardiac life support, return of spontaneous circulation could not be achieved, and death was pronounced 90 minutes after admission. The patient’s clinical course is summarized in Figure 3. 

**Figure 3:**
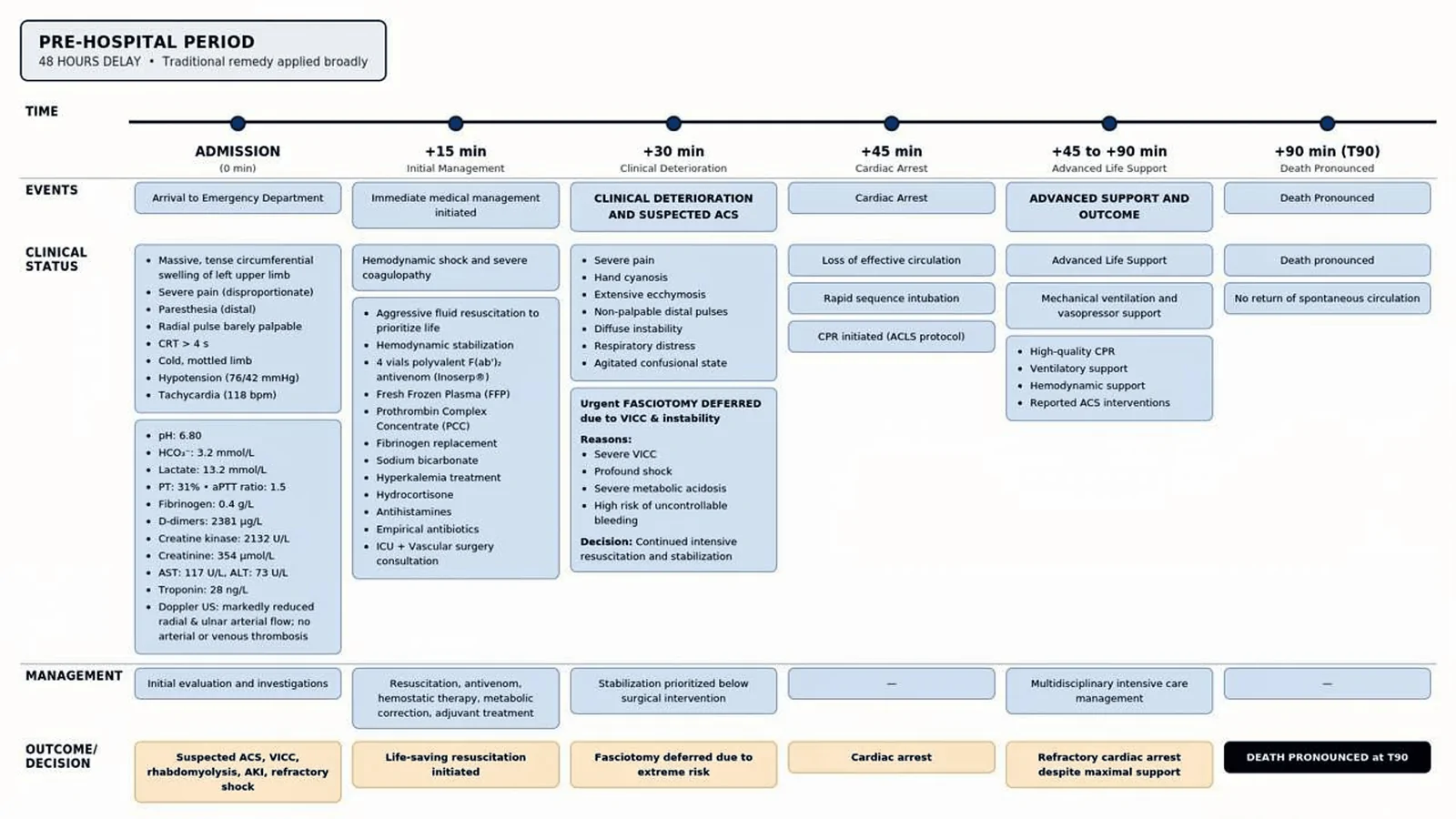
Timeline of the Patient's Clinical Course and Management ACS: Acute Compartment Syndrome; ACLS: Advanced Cardiac Life Support; AKI: Acute Kidney Injury; aPTT: Activated Partial Thromboplastin Time; ALT: Alanine Aminotransferase; AST: Aspartate Aminotransferase; CRT: Capillary Refill Time; CPR: Cardiopulmonary Resuscitation; FFP: Fresh Frozen Plasma; ICU: Intensive Care Unit; PCC: Prothrombin Complex Concentrate; PT: Prothrombin Time; SpO₂: Peripheral Oxygen Saturation; US: Ultrasonography; VICC: Venom-Induced Consumptive Coagulopathy; T90: Time to Death (90 minutes after hospital admission). Image Credits: Figure created by the authors using Python (Matplotlib, version 3.10).

## Discussion

ACS is a limb-threatening surgical emergency resulting from a pathological increase in intracompartmental pressure that compromises tissue perfusion [5]. Although trauma remains its most common cause, ACS is an uncommon but well-recognized complication of snakebite envenomation, particularly following snakebites associated with extensive local tissue injury [8].

Although the offending snake could not be formally identified because it was neither captured nor photographed, Viperidae envenomation was considered highly likely based on the regional epidemiology, the presence of fang marks, rapidly progressive cytotoxic edema, and VICC [2,3,9]. This presentation supported the immediate administration of polyvalent equine F(ab’)₂ antivenom according to current recommendations for severe viper envenomation [9,10].

A notable laboratory finding in our patient was the relative preservation of the platelet count (180×10^9^L) despite severe consumption of coagulation factors, characterized by a prothrombin time of 31%, a fibrinogen level of 0.4 g/L, and markedly elevated D-dimers (2381 µg/L). This dissociation highlights a key pathophysiological distinction between classic disseminated intravascular coagulation (DIC) and VICC. While DIC involves widespread systemic activation of coagulation and secondary thrombin-mediated platelet activation resulting in severe thrombocytopenia, VICC is primarily driven by specific venom procoagulant enzymes (such as thrombin-like enzymes or factor X/prothrombin activators) that rapidly cleave fibrinogen and exhaust clotting factors with relatively limited platelet consumption compared with the factor depletion. Recognizing this distinction is essential for clinicians, as normal platelet levels do not rule out severe, life-threatening coagulopathy following viperid envenomation [4,9].

The diagnosis of ACS after snakebite envenomation remains particularly challenging because extensive venom-induced edema may closely mimic compartment syndrome without a pathological increase in intracompartmental pressure. This entity, often referred to as venom-induced pseudo-compartment syndrome, explains why fasciotomy should not be performed solely on the basis of limb swelling [6,7]. Current recommendations advocate integrating clinical findings with intracompartmental pressure measurements whenever available, particularly when the diagnosis is uncertain. However, pressure-monitoring devices are frequently unavailable in resource-limited settings, forcing clinicians to rely on careful clinical assessment and adjunctive investigations [6].

Although intracompartmental pressure could not be measured in our patient, several findings strongly supported the diagnosis of suspected ACS rather than isolated venom-induced edema. The patient presented with severe pain disproportionate to the visible lesions, pain exacerbated by passive finger extension, diffuse paresthesia, tense circumferential swelling, delayed capillary refill, a cold hand, markedly reduced radial and ulnar arterial flow on Doppler ultrasonography, and biochemical evidence of significant muscle injury with rhabdomyolysis. Nevertheless, definitive confirmation of ACS was not possible because neither intracompartmental pressure measurement nor surgical exploration was performed, and the patient’s family declined a postmortem examination. This diagnostic uncertainty represents one of the principal challenges in the management of severe snakebite envenomation and highlights the importance of interpreting clinical findings within their overall context.

Early administration of antivenom remains the cornerstone of treatment for severe snake envenomation. Neutralization of circulating venom toxins limits systemic toxicity, promotes recovery from VICC, and may reduce progressive local tissue injury, thereby decreasing the risk of secondary ischemic complications [9,10]. In our patient, four vials of polyvalent antivenom were administered immediately after admission. However, because treatment was initiated approximately 48 hours after the bite, extensive local tissue injury and multiple organ dysfunction were already advanced, substantially limiting the potential benefit of antivenom.

The principal therapeutic challenge in this case was not simply whether fasciotomy was indicated, but whether it could be performed safely. When ACS is established or remains highly likely despite appropriate assessment, urgent fasciotomy is the definitive limb-saving intervention [8]. However, in our patient, refractory shock requiring vasopressor support, profound metabolic acidosis, and ongoing VICC, with a prothrombin time of 31% and fibrinogen concentration of 0.4 g/L, made immediate surgical decompression prohibitively hazardous because of the risk of uncontrolled hemorrhage and further hemodynamic deterioration [11,12]. The multidisciplinary team therefore prioritized antivenom administration, hemodynamic resuscitation, correction of coagulopathy, and organ support, with the intention of reassessing the need for fasciotomy after stabilization.

Despite aggressive resuscitation, the patient’s clinical condition deteriorated rapidly, culminating in cardiac arrest 90 minutes after admission. Given the fulminant clinical course, the terminal event was likely multifactorial, driven by refractory shock, severe tissue hypoperfusion with profound lactic acidosis (pH 6.80) and acute venom-induced systemic toxicity. Although progressive limb ischemia and ongoing myonecrosis may have contributed to the overall metabolic and potassium burden [13], the rapid onset of cardiac arrest cannot be attributed primarily to isolated rhabdomyolysis or acute kidney injury [14]. The precise electrophysiological or hemodynamic mechanism of cardiac arrest remains uncertain from the available clinical data. Nevertheless, this case illustrates the devastating synergistic interaction between severe local tissue injury and overwhelming systemic envenomation.

Fatal cases of snakebite envenomation complicated by suspected ACS are rarely reported, largely because timely antivenom administration and, when indicated, surgical management may prevent progression to irreversible local and systemic complications [2,3,5,7]. Our patient, however, presented 48 hours after the bite following initial reliance on traditional treatment, by which time extensive local tissue injury and systemic envenomation had already developed. This substantial delay likely contributed to the severity of the clinical presentation and markedly limited the possibility of reversing the established pathophysiological cascade. Delayed referral is a well-recognized predictor of poor outcome following snakebite envenomation and likely contributed substantially to the rapidly fatal course observed in this case, despite prompt multidisciplinary resuscitation after hospital admission [2,3,5,7].

## Conclusions

This case of suspected ACS highlights the complex diagnostic and therapeutic considerations in severe snake envenomation associated with hemodynamic shock and profound coagulopathy. Delayed medical consultation appears to have facilitated severe tissue damage and coincided with a fatal evolution marked by systemic toxicity, refractory shock, severe metabolic derangement, and acute kidney injury. Surgical decompression was deferred to prioritize critical hemostatic and hemodynamic stabilization. Ultimately, this presentation underlines the importance of early clinical recognition, prompt antivenom administration when indicated, and coordinated multidisciplinary assessment when managing complex envenomation cases.
